# Supplementary material for: Factors Associated with Discrepancy of Child-Adolescent/Parent Reported Quality of Life in the Era of COVID-19
Source: Int J Environ Res Public Health. 2022 Nov 2;19(21):14359. doi: 10.3390/ijerph192114359 (PMC9654617; doi:10.3390/ijerph192114359)
Supplement: Supplementary file 1 [file ijerph-19-14359-s001.zip › ijerph-1959548-supplementary.pdf]

1. *Children and adolescents' HRQoL*

HRQoL was assessed using the self-report and parent proxy versions of the generic 27-item questionnaire, KIDSCREEN-27 [42]. It consists of five HRQoL dimensions for 8 to 18 years old: physical well-being (5 items), psychological well-being (7 items), autonomy and parents (7 items), peers and social support (4 items) and school environment (4 items). Each item was assessed with a 5-modality Likert scale ranging from 0 ("never") to 4 ("always"), reflecting the frequency of behaviors or feelings; or from 0 ("not at all") to 4 ("extremely"), reflecting the intensity of an attitude or belief in the previous week. The item scores were summed following the instructions for scoring the kidscreen-27 and for items formulated with negative response categories (9, 10 and 11), the scores were reversed. For each dimension, a scoring algorithm was used to calculate T-scores scaled with a mean of 50 and a standard deviation of 10. The total standardized score ranged from 0 to 100, with higher scores indicating better HRQoL [42]. These questionnaires have been developed and validated in 13 European countries [7]. The French KIDSCREEN-27 has strong psychometric properties and took less time to complete that the original KIDSCREEN-52 [43].

Parent-reported outcome measures

Parents' self-perceived HRQoL was assessed by the Medical Outcome Study Short Form-12 questionnaire (SF-12) [35]. It is a generic 12-item instrument extracted from the SF-36 questionnaire. A physical health component score (PCS) and a mental health component score (MCS) were calculated from all 12 items. The item scores were summed following the instructions for scoring the SF-12. The total standardized score ranged from 0 to 100, with higher scores indicating better HRQoL. This questionnaire has been validated in 9 European countries and the validity and reliability of the French version have been established [35].

Parents' level of resilience was assessed by the Brief Resilience Scale (BRS). It is a 6-item instrument developed to assess the ability to bounce back or recover from stress [36]. The items are rated on a 5-point Likert scale from 1 ("not at all") to 5 ("completely"). The answers to the items were summed following the instructions for scoring the BRS and for items formulated with negative response

categories (2,4 and 6) the answers to the items were reversed. The total score ranged from 6 to 30, with higher scores indicating higher levels of resilience. Scores ranging from 3 to 4.3 are considered to indicate a normal level of resilience. A score <3 is indicative of low resilience, whereas a score >4.3 indicate high resilience. The French version showed good levels of internal consistency and test-retest reliability, as well as adequate factorial, convergent and discriminant validity [36].

Parents' stress level was assessed by the Perceived Stress Scale-10 (PSS-10) [38]. It is a 10-item instrument derived from the original 14-item form developed to assess "the degree situations in one's life appraised as stressful". Parents were asked to indicate the frequency of an indicator of stress over the past month with each item on a 5-point Likert scale ranging from 0 ("never") to 4 ("very often"). The item scores were summed following the instructions for scoring the PSS and for items formulated with negative response categories (4, 5, 7 and 8), the scores were reversed. The total score ranged from 0 to 40, with higher scores indicating higher levels of perceived stress. Total mean scores of 0-13 indicate low stress, scores of 14-26 indicate moderate stress, and scores of 27-40 indicate high stress [37]. A good internal consistency, with a Cronbach's alpha coefficient of 0.83 and reliability of the French version have been established [38].

Parents' anxiety symptoms were assessed by the 7-item Generalized Anxiety Disorder Scale (GAD-7) [39] as it is currently the most widely used anxiety measure, both in clinical practice and research . It is a 7-item instrument based on seven core symptoms. Parents were asked to indicate how often they were bothered by each symptom during the last two weeks with each item on a 4-point Likert scale ranging from 0 ("not at all") to 3 ("nearly every day"). The total score ranged from 0 to 21 with higher scores indicating higher levels of anxiety. The scores for symptom severity were 0-4 for normal anxiety, 5-9 for mild anxiety, 10-14 for moderate anxiety, and 15-21 for severe anxiety. Good internal consistency, with Cronbach's alpha coefficient of 0.898, and good external validity have been established [39].

Parents' self-perceived social support was assessed by the Multidimensional Scale of Perceived Social Support (MSPSS) [40]. It is a 12-item instrument that assesses social support from three sources: family, friends and significant others [41]. Parents were asked to indicate their level of agreement with each item on a 7-point Likert scale ranging from 1 ("very strongly disagree") to 7 ("very strongly agree").

The total score of each dimension ranged from 1 to 7 with higher scores indicating higher perceived social support. Good internal reliability and reproducibility of the French version have been established [40].

Socio-demographic data, living and learning conditions during and after lockdown

Children-adolescents completed a self-administered questionnaire that included sociodemographic data (age, gender, educational level) and living (frequency of exiting home) and learning conditions (time spent on schoolwork at home, focus at home for homework, difficulty isolating at home, tensions and conflicts at home and with neighbors, noises inside and outside the residence). Parents completed a self-administered questionnaire that included sociodemographic measures such as gender, living arrangements, home location and living conditions such as having a person infected with COVID-19 at home.
